# Green Polymer Approach in the Production of Polyurethane Foams Using Chitosan and Citric Acid-Based Polyols

**DOI:** 10.1021/acsomega.6c03826

**Published:** 2026-07-27

**Authors:** Royal Guliyev, Nalan Tekin, Mustafa Özgur Bora, Yavuz Emre Yağcı, Nizami Shamiyev

**Affiliations:** † 52980 Kocaeli University, Faculty of Arts and Science, Department of Chemistry, Kocaeli 41001, Turkey; ‡ Kocaeli University, Faculty of Aeronautics and Astronautics, Aviation Material Research and Development Laboratory, Kocaeli 41380, Turkey; § Farplas Otomotiv A.S., Taysad Organize Sanayi Bölgesi (TOSB), Kocaeli 41420, Turkey; ∥ 522675 Eskişehir Technical University, Faculty of Science, Department of Chemistry, Eskişehir 26140, Turkiye

## Abstract

Polyurethane (PU) foams produced from fossil-based raw materials pose significant environmental and economic challenges. Consequently, sustainable and eco-friendly production methods have become a focal point for researchers in recent years. In this study, the potential use of chitosan as an alternative to fossil-based polyols was investigated in detail. Unlike methods described in the literature, it has been demonstrated that chitosan can be liquefied using citric acid, a low-cost and environmentally friendly component. MALDI-TOF MS and FTIR analyses, when evaluated together, confirmed that chitosan forms new bonds through an esterification reaction. The resulting biopolyol was used for PU foam synthesis by replacing 50% of the fossil-based polyether polyol. The chemical structure, morphology, and mechanical and thermal properties of the produced foams were evaluated using Fourier-transform infrared (FTIR) spectroscopy, scanning electron microscopy (SEM), compressive strength, and thermogravimetric analysis (TGA), respectively. The results showed that the maximum decomposition temperature (75% mass loss) of PU foams produced using the chitosan-based biopolyol liquefied with citric acid increased from 390 to 429 °C. These findings demonstrate that the proposed method offers an alternative that is both environmentally friendly and economically beneficial. Furthermore, the fact that this approach to biopolyol production via the liquefaction of chitosan with citric acid is reported for the first time in the literature underscores the innovative nature of the study.

## Graphical abstract

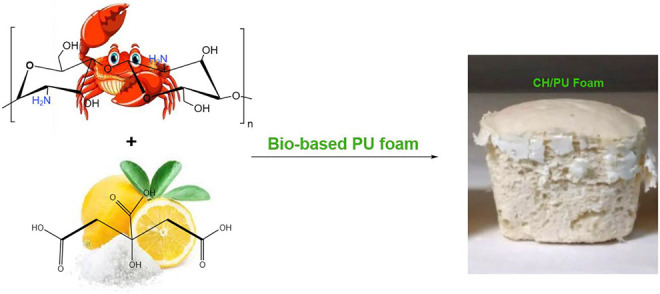

## 1 Introduction

Polyurethane foams (PUF) are a significant class of polymers utilized in industries such as sound insulation, thermal insulation, textiles, construction, and automotive manufacturing. Their production relies on polyols, including polyether polyols and polyester polyols, and isocyanates. Mixing these liquid components in small quantities yields a high-volume, easily applicable material.^1–3^ This characteristic has contributed to increased demand for PU foams and has expanded their global market share annually.^4^ However, reliance on fossil-based raw materials causes volatility in cost and performance. Furthermore, the manufacturing process involves intensive chemical procedures and offers limited recycling options, resulting in substantial environmental concerns.^5,6^

For this reason, research into polyurethane (PU) foam production using polyol-like biopolymers obtained from sustainable sources has gained momentum in recent years. Emphasis is being placed on replacing the basic chemicals used in PU production, especially polyols, with biopolymer derivatives enriched with hydroxy (−OH) groups. This approach is considered a more promising strategy for reducing the environmental problems caused by fossil-based raw materials.^7^

To this end, various studies have been conducted on the partial replacement of fossil-based polyols with biobased alternatives to produce polyurethane (PU) foam. In one study, flexible PU foams were produced by reducing the amount of fossil-based polyol from 15% to 35% using a biopolyol obtained through the epoxidation of olive oil.^8^ Although this approach contributes to sustainability, the cost-performance evaluation of the base raw material indicates that there is still a need for more cost-effective biobased sources. Hu and Li (2014) produced biobased polyols with a high hydroxyl value (OH number: 536–936 mg KOH/g) by processing lignocellulosic biomass using a two-stage liquefaction method (an acid-catalyzed first stage and a base-catalyzed second stage) involving crude glycerol. Polyurethane foams produced using the obtained polyols exhibit suitable mechanical and insulating properties, such as low thermal conductivity and high compressive strength. However, it has been noted that the high viscosity and compositional heterogeneity of the obtained polyols may present certain challenges in process control.^9^ In this study, Kosmela et al. (2018) systematically investigated the use of innovative biopolyols obtained by liquefying cellulose biomass in the presence of crude glycerol, a sustainable byproduct of biodiesel production, possessing a high hydroxyl value (754 mg KOH/g) in the synthesis of rigid polyurethane foam. In a system where petrochemical polyol was replaced by this biopolyol at a ratio of 0–70% by weight, the authors observed that an increasing proportion of the biocomponent refined the cellular morphology of the foams, thereby enhancing their mechanical strength. However, the researchers found that the addition of this highly functional biopolyol led to a decrease in the thermal stability of the matrix.^10^ In another study, PU foams were produced by replacing the fossil-based polyol with kraft lignin and castor oil at ratios of 5–20% and 10–100%, respectively. The findings revealed that the compressive strength of the lignin-containing foams was significantly lower than that of the control sample, while the use of castor oil caused expansion in the cell pores, negatively affecting the compressive strength.^11^ Similarly, Tran et al. (2022) investigated the potential of bio-oil obtained from sugarcane bagasse using the hydrothermal liquefaction (HTL) method with glycerol as a cosolvent as an alternative raw material for biobased PU foam production. This study evaluated the effects of HTL parameters, such as glycerol loading (0–50 wt %) and alkaline catalyst concentration (0.05–1 M K_2_CO_3_), as well as the replacement of polyol with bio-oil (0–100 wt %) on the thermal stability and mechanical properties of the foams. The results indicate that PU samples containing bio-oil exhibit reduced thermal stability, but those containing 50% bio-oil demonstrate the highest compressive strength.^12,13^ Additionally, it has been reported that commonly used methods such as epoxidation or chain opening are applied to produce biobased polyols from vegetable oils.^11^ However, these methods are typically two-step processes that require significant time and effort. Additionally, the extensive use of these oils in the food chain has been reported to cause price increases, leading to competition in raw material supply.^14–16^

Chitosan is a biocompatible and water-soluble compound obtained by the deacetylation of chitin; a polysaccharide found abundantly in nature. Owing to its biocompatibility and low cost, its potential as a solution has been evaluated in various studies.^17,18^ In recent years, chitosan has also been used in the production of polyurethane (PU) foams. In these applications, some studies have aimed to improve the mechanical and thermal properties of foams by using chitosan as a filler material,^19,20^ while others have evaluated the potential of using chitosan as a substitute for polyol.^21,22^ In this study, Strzałka and Lubczak (2023) synthesized biobased polyols by hydroxyalkylating chitosan with glycidol and ethylene carbonate, and the resulting polyols were subsequently used to produce rigid polyurethane foams.^23^ This study provides important insights into the use of chitosan for PU foam production. Nevertheless, chemicals such as glycidol^24^ and ethylene carbonate^25^ present several disadvantages concerning health and environmental impact. Additionally, from an economic perspective, the market price of ethylene carbonate ranges from approximately 1.16 to 1.59 USD/kg,^26,27^ whereas glycidol is valued at approximately 20 USD/kg,^28^ highlighting their economic constraints. Consequently, there is a recognized need to develop alternative materials that can substitute these components to mitigate environmental and health risks while enhancing cost-effectiveness. Compared to these chemicals, citric acid is considered more environmentally safe^29^ and economically viable.^30,31^

This study aims to increase the potential for the use of biopolymers derived from renewable sources in industrial applications by converting chitosan into polyols via environmentally friendly methods. Instead of liquefaction agents such as glycidol and ethylene carbonate, which are widely used in the literature but are environmentally problematic, citric acid (lemon salt) and glycerin have been preferred as alternatives in line with the sustainability approach. In this context, chitosan was processed in a 25% citric acid solution to prepare a biobased polyol. The potential application of the obtained biopolyol in polyurethane foam (PU) production was investigated, and the maximum substitution ratio that could maintain semirigid PU foam properties was determined. In this study, up to 50% of the fossil-based polyol was substituted with a biobased polyol. The biobased polyol-containing polyurethane foam samples produced at this ratio were comprehensively evaluated in terms of apparent density, thermal stability, surface morphology, mechanical strength (compression test), and combustion behavior, and compared with the control samples. The results demonstrate that chitosan-based biopolyols can serve as an environmentally sustainable alternative to fossil-based polyols in the production of semirigid polyurethane foam.

## 2 Experimental Section

### 2.1 Materials

A three-functional polyether polyol (density 1.02 g/cm^3^ and viscosity 1470 mPa.s at 25 °C; Mn ≈ 3000 g/mol; OH number 57) and polymeric methylene diphenyl diisocyanate (PMDI, density 1.21 g/cm^3^ and viscosity 155 mPa·s; NCO content ≈ 31.5%; functionality ≈ 2.8) were supplied by Farplas Otomotiv A.Ş., a local company. Glycerin (99.9%), sulfuric acid (H_2_SO_4_), and sodium bicarbonate (NaHCO_3_) were obtained from Sigma-Aldrich, while the citric acid used as lemon salt was purchased from the market. The dibutyltin dilaurate (DBTDL) catalyst and surfactant were supplied by Kimpur. Chitosan, possessing a molecular weight between 50 and 350 kDa and a deacetylation degree of 90%, was obtained from Biyopol Company.

### 2.2 Preparation of Chitosan-Based Polyol

To increase the citric acid concentration in water to approximately 25%, 83.4 g of lemon salt was added to distilled water. Subsequently, 15 g of chitosan was added to a 250 mL container containing approximately 25% citric acid. The mixture was stirred for 3 h at 120 °C using a magnetic stirrer at a rotation speed of 250 rpm.

The mixture was then cooled to 100 °C, and 50 mL of glycerin and 0.5% sulfuric acid (H_2_SO_4_) were added as a catalyst. The mixture was stirred at 250 rpm for 3 h. This process yields a chitosan-based polyol. H_2_SO_4_ in the polyol was neutralized using sodium bicarbonate (NaHCO_3_). The predicted chemical mechanism for the formation of chitosan-based biopolyol is presented in Figure 1.

**Figure 1:**
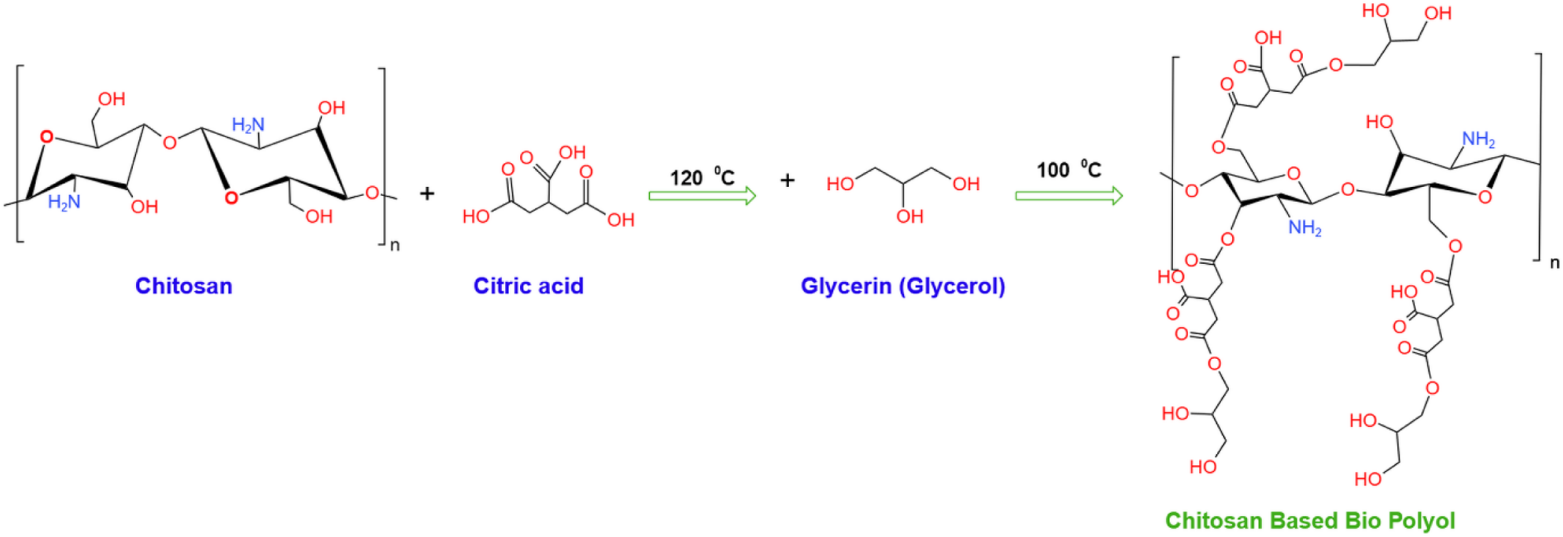
Mechanism representing the formation of chitosan-based biopolyol.

### 2.3 Preparation of Chitosan-Based Polyurethane Foam

As shown in Table 1, the amount of fossil-based polyether polyol was reduced by 25%, 50%, and 65% using biobased polyol derived from chitosan instead of polyether polyol.

**Table 1:** Formulations of Standard and Chitosan-Based Polyurethane Foams Based on 100 g of Polyol^a^

Sample	Polyether Polyol (g)	Chitosan-Based Polyol (g)	Isocyanate (g)	DBTDL (g)	H_2_O (g)	Surfactant (g)
Standard PU Foam	100	0	65	1.5	1.5	0.75
25 CH/PU Foam	75	25	65	1.5	1.5	0.75
50 CH/PU Foam	50	50	65	1.5	1.2	0.75
65 CH/PU Foam	35	65	65	1.5	1.2	0.75

a Note: In polyurethane foam formulations containing 50% and 65% chitosan-based polyol, the amount of water added to the system was optimized to 0.71% by weight, considering the trace amounts of residual water that may be retained in the structure via hydrogen bonds during polyol synthesis.

The formulation presented in Table 1 was used as the basis for producing biobased polyol-based polyurethane (PU) foams. In the first stage, chitosan-based polyol, polyether polyol, a foaming agent (H_2_O), 1% (by weight) dibutyltin dilaurate (DBTDL) catalyst, and 0.5% (by weight) surfactant were added to a reaction vessel, and the mixture was stirred at 1000 rpm for 40 s to ensure homogenization.

The partial replacement of fossil-based polyols with biobased polyols significantly affected the reaction kinetics and rheological behavior of the polyurethane foam system. The results regarding the characteristic process parameters, such as the onset of foaming, free rise time, and gelation time, are presented in Table 2. The results demonstrated that as the proportion of biobased polyol in the formulation increased, the reaction rate decreased, leading to a significant increase in the free rise and gelation times.

**Table 2:** Foaming Properties of Standard and Bio-Based Polyurethane Foams

Sample	Cream Time (s)	Foaming Start Time (s)	Free Rise Time (s)	Gel Time (s)	NCO/OH Ratio
Standard PU Foam	11	35	83	189	1.82
25 CH/PU Foam	17	53	94	230	0.88
50 CH/PU Foam	19	67	105	235	0.61
65 CH/PU Foam	37	142	247	650	0.50

Consequently, as shown in Figure 2, the addition of a chitosan-based polyol caused the PU foam structure to lose its flexibility and acquire a more rigid character.

**Figure 2:**
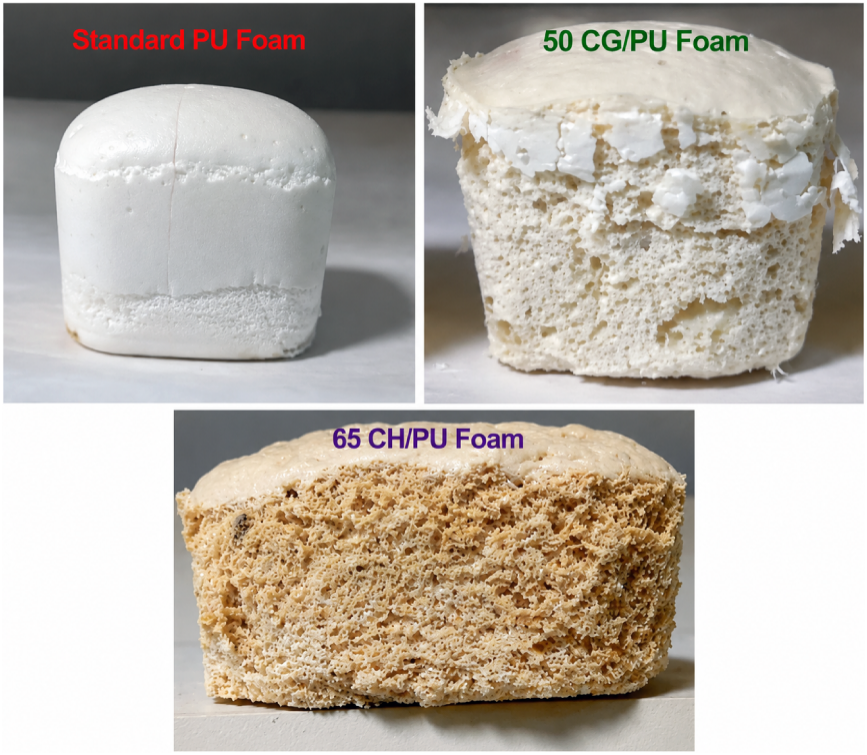
Images of standard PU and CH/PU foam samples have been prepared.

The prepared reaction mixture was left to stand at room temperature for approximately 24 h to complete the curing process. The resulting standard PU and CH/PU foam samples are illustrated in Figure 2. Increasing the proportion of chitosan-based polyol in the formulation significantly affected the foam formation. In particular, when the chitosan-based polyol content was increased to above 50%, delays in the foaming reaction and significant reductions in foam volume were observed. Therefore, the biobased polyol substitution ratio was limited to 50% in this study. However, a foam sample containing 65% chitosan-based polyol was prepared for comparative evaluation. As shown in Figure 2, the foam structure became irregular, and the volumetric expansion decreased significantly in this sample. Additionally, the foam containing 65% chitosan-based polyol exhibited an excessively brittle structure, and its mechanical integrity was significantly weakened.

Note: In this study, biopolyol contents of 25%, 50%, and 65% were evaluated in the formulations. Based on preliminary experimental studies and process optimizations, it was determined that a 50% biopolyol content provided the most balanced and reproducible performance in terms of foam formation and material homogeneity. Although the effect of biobased content was observed to be limited at lower concentrations, polyurethane foams produced with 65% biopolyol content exhibited a distinctly brittle structure, with a significant decrease in mechanical strength and weakening of structural integrity. This finding indicates that a high biobased content negatively affects the continuity of the foam network. Therefore, detailed characterization analyses were conducted exclusively on the 50% biopolyol content, which demonstrated optimal performance.

### 2.4 Characterization

The hydroxyl (OH) and acid numbers of the prepared biobased polyol were determined in accordance with the ASTM D4274-11 and ASTM D4662-08 standards specified in the literature.^32^ For this purpose, an analytical titration method was applied using a methanol solution containing 0.1 M KOH. Phenolphthalein was selected as the indicator.

To determine the molecular weight distributions of the biobased polyol prepared in this study, a Bruker Daltonics Matrix-Assisted Laser Desorption/Ionization Time-of-Flight mass spectrometer (MALDI-TOF MS) was used, and the analyses were performed in positive ion mode.

GPC (Gel Permeation Chromatography) analysis was performed to determine the molecular weight distribution of the biobased polyol. Prior to the analysis, 5 mg of the biobased polyol was dissolved in 3 mL of tetrahydrofuran (THF), and the resulting solution was injected into the GPC system.

Compression tests were performed on specimens prepared with dimensions of 30 mm × 30 mm × 30 mm. The semistatic compression behavior of the specimens was evaluated using an electronic universal testing machine (CMT4304-QY, MTS Systems Corporation, Minnesota, USA) at a strain rate of 0.001 s^–1^. The tests were terminated when the specimens reached a strain level of 70%. To ensure the reliability and reproducibility of the results, each test was carried out in five replicates, and the average values obtained were reported as the final test results.

To investigate the structural characterization of standard PU, and CH/PU foam samples, FTIR spectra were obtained using Diamond ATR on a Bruker Tensor 27 spectrophotometer at 4000 to 400 cm^–1^ with 30 scans and 2 cm^–1^ resolution.

UL 94 Vertical Burning (VB) tests were performed on standard PU, and CH/PU foam samples measuring 80 mm × 20 mm × 10 mm (length × width × thickness). Five repeated tests were performed for each sample.

The thermal behavior of standard PU, and CH/PU foam samples was determined by thermal gravimetric analysis (TGA; Mettler Toledo TGA 1) at a heating rate of 10 °C/min from 25 to 600 °C under an N_2_ atmosphere.

To obtain information about the surface of the standard PU, and CH/PU foam samples, analysis was performed using a JEOL JSM-6060 scanning electron microscope (SEM). The SEM was silver-coated and operated with a secondary electron detector to obtain detailed images of the surface morphology.

The apparent densities of the foam samples were determined using ASTM D1622 in accordance with ASTM D1622 using samples with dimensions of 20 mm × 20 mm × 10 mm (length × width × thickness). The dimensions and masses of the samples were determined using calipers and electronic scales, respectively. The apparent density of each sample was calculated by determining the mass-to-volume ratio in five replicates, and the average values obtained were expressed in g/cm^3^.

A short-term water absorption test lasting 72 h was conducted to investigate the water absorption behavior of the standard PU and CH/PU foam samples. Five replicates were prepared for each formulation, and the samples, prepared to a dimension of approximately 30 mm × 30 mm × 20 mm, were first dried in an oven at 75 °C for 24 h. After drying, the samples were weighed, and their initial masses (M1) were recorded. The samples were then placed in a container filled with water to ensure complete submersion. To ensure that all surfaces of the samples came into equal contact with the water and remained stationary, a glass plate of a specific mass was placed on top of the samples and secured using its thin edge. After a 72-h immersion period, the samples were removed from the water, and any remaining free water droplets on their surfaces were carefully wiped off with paper towels. The final masses (M2) were recorded. The water absorption capacities of the samples were calculated using the initial and final mass values via eq 1 below:^33,34^

1 $$\mathrm{Water Absorption}\;\left(\%\right)=\left(\frac{M2-M1}{M1}\times 100\right)$$

where;

• M_1_: The initial dry mass of the sample,

• M_2_: The mass measured after immersion in water.

## 3 Results and Discussion

### 3.1 Hydroxyl and Acid Values of Bio-Based Polyol

The acid and hydroxyl numbers of the biobased polyol, synthesized primarily from chitosan, citric acid, and glycerol, were determined by titrimetric analysis using a 0.1 M KOH/methanol solution and phenolphthalein indicator. The calculations were based on the volume of KOH consumed during titration. According to the analysis results, the acid number was approximately 5.8 mg KOH/g. This higher value compared to that of fossil-derived polyether polyols can be explained by the presence of residual acidic groups arising from the multifunctional carboxylic structure of citric acid. Furthermore, the high hydroxyl number of ∼695 mg KOH/g indicates an increase in the density of the functional groups within the system.

### 3.2 Molecular Weight Determination by MALDI-TOF MS

MALDI-TOF MS was employed to determine the molecular weight distribution, number-average molecular weight (Mn), and polydispersity index of the biobased polyol obtained.^22,35^ As a significant portion of the synthesized polyol consisted of chitosan derivatives, MALDI-TOF MS analyses were performed on both pure chitosan and the biobased polyol obtained using citric acid, glycerol, and chitosan to carry out a comparative evaluation. The analysis results for chitosan are presented in Figure 3a and Table 3, respectively.

**Figure 3:**
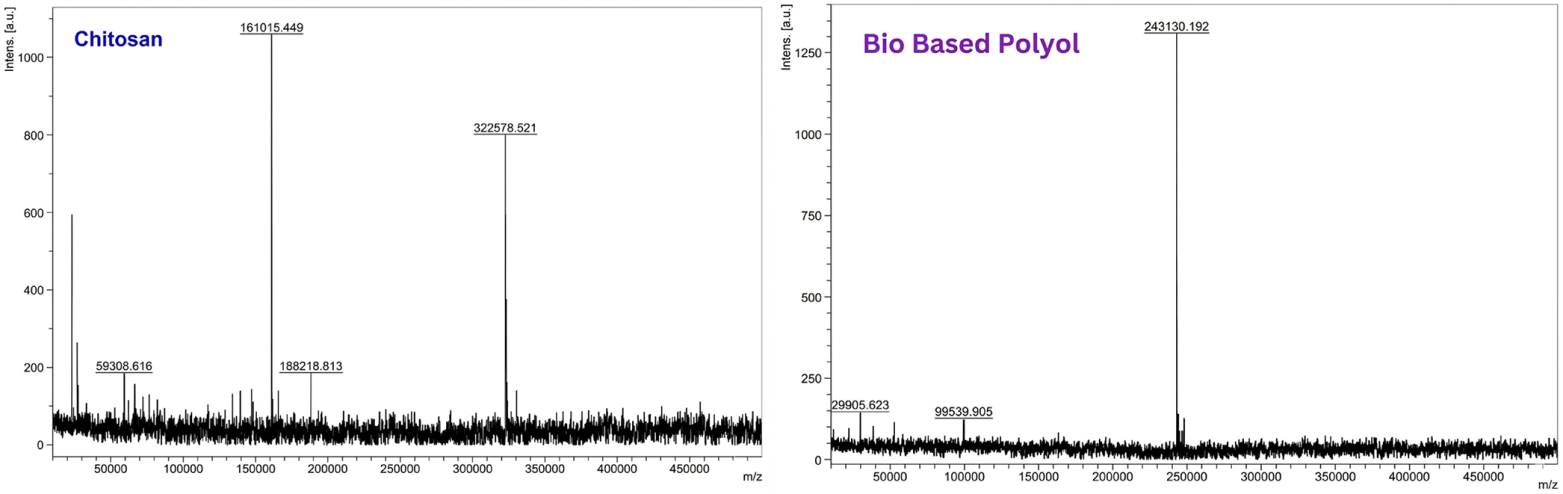
Graphs relating to the MALDI-TOF MS analysis of (a) chitosan and (b) biobased polyol.

**Table 3:** Calculated MALDI-TOF MS Mass Distribution and Polydispersity Data for Chitosan

*m/z* (*Mi*)	Signal Intensity (*Ni)*	Mn (Da) $\left(\frac{\Sigma NiMi}{\Sigma Ni}\right)$	Mw (Da) $\left(\frac{\Sigma NiMi^{2}}{\Sigma NiMi}\right)$	Dispersity Indices (Đ) $\left(\frac{Mw}{Mn}\right)$
23123.248	594	34,260.2	42,336.5	1.23
26871.271	263			
27174.104	153			
59308.616	183			
66483.485	157			
147257.215	143	162,126.1	162,482.0	1.002
161015.449	1056			
161031.392	519			
161034.352	520			
161077.605	191			
161133.499	141			
188218.813	183			
322578.521	798	322,619.7	322,641.8	1.00
322596.474	650			
322612.274	667			
322664.503	251			
322680.362	234			
322742.260	226			
322824.931	265			
322845.839	232			
322937.294	284			
322956.166	304			
322967.651	285			
322988.144	236			
323078.090	340			
323093.247	375			
323512.807	160			
323523.173	162			

The data obtained indicate that the molecular weight distribution of pure chitosan consists primarily of macromolecular fractions concentrated at approximately 161 and 322 kDa. A marked increase in the signal intensity in these regions is clearly observable in both the tabulated data and Figure 3a, indicating that chitosan possesses a multimodal molecular weight distribution.

This situation stems from the natural origin and partially heterogeneous structure of chitosan, indicating the coexistence of oligomeric units with different chain lengths. The results confirmed that chitosan possesses a broad, multimodal molecular weight distribution rather than a narrow, uniform polymer. As shown in Table 3, the mass distribution of chitosan generally clustered into three distinct, closely spaced ranges. The first of these ranges varied between 23 and 66 kDa, the second and most dominant distribution lay within the 147–188 kDa range, and the third distribution was situated around 322 kDa. As the direct calculation of general Mn and Mw values is difficult in systems exhibiting this type of macromolecular branching, the average Mn and Mw values were calculated by taking these three closely related distributions into account, based on the work of Aksenov et al. (2008)^36^ and other literature studies.^37,38^

When the MALDI-TOF MS results presented in Table 4 and Figure 3 are evaluated together, it can be seen that the macromolecular weight distribution in the biobased polyol is concentrated within a narrower range. A dominant peak distribution was observed in the spectrum at approximately 243 kDa, indicating that most of the signal intensity was concentrated in this region. Consequently, the polydispersity index, based on the number-average molecular weight (Mn) and weight-average molecular weight (Mw) values listed in Table 4, were calculated using this molecular weight range as a basis. In contrast, the peaks at approximately 322 and 161 kDa observed for chitosan in Table 3 were significantly reduced or undetectable in the biobased polyol.

**Table 4:** Calculated MALDI-TOF MS Mass Distribution and Polydispersity Data for the Bio-Based Polyol

*m/z* *(Mi)*	Signal Intensity (*Ni)*	Mn (Da) $\left(\frac{\Sigma NiMi}{\Sigma Ni}\right)$	Mw (Da) $\left(\frac{\Sigma NiMi^{2}}{\Sigma NiMi}\right)$	Dispersity Indices (Đ) $\left(\frac{Mw}{Mn}\right)$
29905.623	140	≈243,579.9	≈243,587.2	≈1.00
52831.797	114			
99539.905	122			
99549.816	118			
243130.192	1306			
243939.448	126			
243951.519	138			
243959.528	128			
248191.315	126			

These findings suggest that chitosan undergoes depolymerization during liquefaction, followed by the reassociation of lower-molecular-weight species to form a more homogeneous distribution. However, it should be noted that this interpretation does not provide direct evidence of the mechanism and is merely an indirect assessment based on the MALDI-TOF data. Furthermore, citric acid may act as a potential cross-linking agent in this process.

### 3.3 GPC (Gel Permeation Chromatography) Analysis

Gel Permeation Chromatography (GPC) analysis was performed using tetrahydrofuran (THF) as the solvent to determine the molecular weight and chain distribution of the chitosan-based biopolymer obtained. According to the analysis results, the number-average molecular weight (Mn) was 47.063 g/mol, and the weight-average molecular weight (Mw) was 230.219 g/mol. In contrast, the Mn and Mw values calculated from the experimental MALDI-TOF MS spectral data were 243.579 and 243.587 g/mol, respectively.

The Mw values of the macromolecules were similar in the GPC and MALDI-TOF analyses. The Mw value determined by GPC was 230.219 g/mol, and the Mw value calculated from the MALDI-TOF analysis was 243.578 g/mol, indicating that the dominant molecular weight region of the synthesized biopolymer was defined consistently and similarly by both techniques. In contrast, a significant deviation was observed between Mn values. The Mn value recorded in the GPC analysis was 47.063 g/mol, whereas that calculated from the MALDI-TOF data was 230.570 g/mol.

The primary reason for this difference is that the physical and chemical measurement principles underlying molecular weight determination in the two methods are entirely different from each other. The GPC technique separates macromolecules based on their hydrodynamic volumes in solution and determines the molecular weight relatively using linear calibration standards (polystyrene).^39^ In particular, the highly branched (hyperbranched) structures found in chitosan-based modified biopolymers, dense spherical morphology, and different chain architectures resulting from citric acid/glycerol reactions significantly alter the hydrodynamic behavior of polymer chains in solution. Because branched structures occupy a smaller volume in the solvent than linear standards, the GPC method tends to underestimate the molecular weight. Furthermore, the presence of oligomeric fractions that remain unreacted in the system or are short-chain disproportionately lowers the calculation of Mn, which is a number-average parameter.^39,40^

In contrast, MALDI-TOF MS is an absolute method that directly identifies molecular species based on their mass-to-charge (*m*/*z*) ratios, independent of the solution volume. Average mass calculations in MALDI-TOF analyses were performed using the main oligomer/polymer series, which exhibited high ionization tendencies and dominant intensities in the spectrum. Owing to the “mass discrimination” effect known in polymer chemistry or the selective ionization dynamics in the matrix environment, free byproducts present at low concentrations or very short-chain fractions may not produce signals as strong as the main macro-chains in the MALDI-TOF spectrum. Consequently, while the MALDI-TOF method reveals the absolute average molecular weight representing the dominant macromolecular population in the system, the GPC method accounts for the entire eluting distribution as well as small oligomeric tails.^37–42^

The polydispersity index (PDI) was calculated from the GPC data as follows (2):

2 $$PDI=\frac{Mw}{Mn}=\frac{230.219}{47.063}=4.89$$

This high value confirms that the molecular weight distribution of the synthesized biopolyol is quite broad, that polymeric species with different chain lengths are formed in the reaction medium, and that heterogeneous branching reactions caused by multifunctional monomers have occurred.

The polydispersity index calculated from the MALDI-TOF spectrum data was 1.00. This narrow distribution indicates that the main series emitting signals and ionizing in the MALDI-TOF spectrum form a homogeneous population with molecular weights that are extremely close to one another. It is known from the literature that in complex and branched biobased polymer systems, GPC provides a broad macrodistribution encompassing all intermediates, whereas MALDI-TOF selectively represents the main polymeric backbone on an absolute mass basis.^41,42^ Consequently, when the data obtained from both methods were evaluated together, it was successfully demonstrated that the synthesized chitosan-based biopolyol contained a dominant, high-molecular-weight, and stable main fraction, along with low-molecular-weight oligomeric components that extended the overall matrix structure.

### 3.4 Fourier Transform Infrared Spectroscopy (FTIR)

The chemical structural properties of the biobased polyol obtained by modifying chitosan with citric acid and glycerin were examined in detail using FTIR spectroscopy. The FTIR spectra of chitosan and biobased polyol samples are presented in Figure 4a. Upon evaluation of the spectra, the broad bands observed in the 3300–3550 cm^–1^ wavenumber range were attributed to the stretching vibrations of the −NH and −OH groups. The fact that these bands exhibit a broader and more intense character in the biobased polyol indicates an increase in the hydroxyl group concentration and, consequently, strengthened hydrogen bonding interactions. This indicates that the density of functional groups in the system increased as a result of structural modification. The peaks observed at 2963 and 2868 cm^–1^ are associated with the symmetric and asymmetric stretching vibrations of aliphatic −CH groups.^21,22^ Additionally, the peak at 1028 cm^–1^ corresponds to C–O stretching vibrations, while the prominent band at 1743 cm^–1^ indicates the presence of carbonyl (C=O) groups.^19,43^ The vibrational band observed at 1559 cm^–1^ is attributed to the in-plane N–H bending (Amide II) vibrations of the amino and residual amide groups present in the chitosan structure. Overall, no significant shift in the position or loss of intensity of this band was observed following the modification process, indicating that the nitrogen-containing functional groups in the chitosan backbone were largely chemically preserved. This stability in the amine/amide regions suggests that the modification reaction with citric acid proceeded selectively via the hydroxyl (−OH) groups in the chitosan structure rather than through the amine groups. In contrast, the carbonyl (C=O) stretching band at 1743 cm^–1^ associated with ester and carboxylic acid structures—appeared quite distinct, sharp, and intense in the modified samples.^43,44^ While the absorption band in this region is weaker in the spectrum of pure chitosan, this immense increase in the intensity and sharpness of the 1743 cm^–1^ band after modification clearly demonstrates the formation of new chemical moieties in the system. These spectral observations confirm that esterification reactions between chitosan and citric acid, a multifunctional carboxylic acid, occurred successfully.

**Figure 4:**
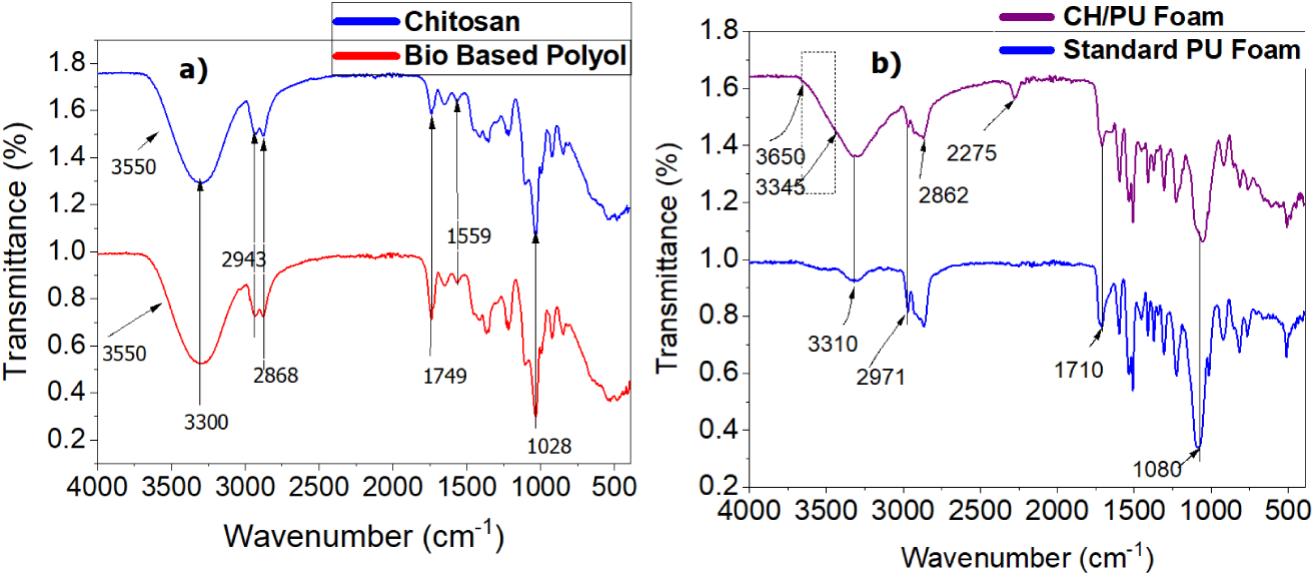
FTIR spectra of (a) chitosan and biobased polyol (b) CH/PU foam and standard PU foam.

The ester bonds formed as a result of the dehydration reaction between the primary and secondary hydroxyl (−OH) groups of chitosan and the carboxylic acid groups of citric acid are the primary source of the sharpening and increase in intensity in the 1743 cm^–1^ region. Furthermore, the successful integration of citric acid into the structure, along with the increased density of carbonyl functional groups within the matrix, directly supports this finding. Consequently, the FTIR data demonstrate that esterification is the predominant reaction pathway in the synthesis of chitosan-based biopolyols.

A comparative analysis of the spectra of chitosan and biobased polyols showed that significant changes occurred in the distribution of functional groups depending on the modification process. The increase in the bandwidth in the 3300–3550 cm^–1^ range indicates an increase in the number of hydroxyl groups and intensified hydrogen bonding interactions. Meanwhile, the increase in the peak intensity at 1743 cm^–1^ confirms the formation of carbonyl-containing structures. When these findings are evaluated together, it is understood that glycerin is bound to the chitosan chain via citric acid, and that an esterification reaction occurs during this process.

The results showed that chemical modification resulted in an increase in both hydroxyl and ester functional groups in the biobased polyol structure, thereby enhancing the functional group diversity and reactivity of the material. These structural characteristics indicate that the obtained biobased polyol possesses a suitable chemical framework for use in advanced material applications, particularly in polyurethane.

The chemical changes in the structure were examined using FTIR analysis performed on standard PU foam and CH/PU foam samples, and the spectrum results obtained are presented in Figure 4b. The peak observed at a wavelength of 3310 cm^–1^ corresponds to the stretching vibrations of the NH groups. The peaks at 2971 and 2862 cm^–1^ correspond to the vibrations of asymmetric and symmetric CH groups, respectively.^23^ Additionally, the absorption bands identified at 1710 and 1080 cm^–1^ correspond to the vibrations of the C=O and C–O bonds, respectively.^43^ These findings indicate that the structural properties of the CH/PU sample containing 50% CH-based polyol were highly like those of the standard PU sample.

However, the absorption band observed at a wavenumber of 2275 cm^–1^ indicates the presence of NCO groups.^45^ This suggests that the interaction between the −OH and −NCO groups is not complete and that free isocyanate groups remain in the structure. Additionally, the broad band detected in the 3650–3345 cm^–1^ range indicates that free −OH groups are present in significant amounts in the CH/PU structure.^46,47^ This phenomenon stems from the tendency of the high concentration of −OH groups in CH-based polyols to form strong H-bonds. This intense hydrogen bonding causes a rapid increase in gel viscosity within the system, thereby limiting the effectiveness of the interactions between the remaining and OH and −NCO groups in the reaction medium. Additionally, it is believed that the hydrogen bonds formed between free and OH groups kinetically slow down their complete and effective reaction with the −NCO groups.^47^ This situation restricts the reaction progression, leading to the formation of a more densely cross-linked structure and, consequently, contributing to the resulting foam exhibiting a harder and stiffer morphological profile compared to that of the reference semirigid polyurethane foam.

### 3.5 Thermal Behavior

Thermogravimetric analysis (TGA) was used to obtain information about the thermal behavior of the CH-based PU foam and standard PU foam samples. The TGA/DTG curves shown in Figure 5 provide information about the thermal stabilities of the CH/PU foam and standard PU foam samples. The mass losses at T%5, T%10, T%20, T%50, and T%75 shown in the graphs are presented in more detail in Table 5.

**Table 5:** DTG Values for Standard PU and CH/PU Foam Samples

Sample	T%5 (°C)	T%10 (°C)	T%20 (°C)	T%50 (°C)	T%75 (°C)	Char Yield (%)
Standard PU Foam	266	286	316	368	390	5.4
CH/PU Foam	110	170	230	366	429	14.7

**Figure 5:**
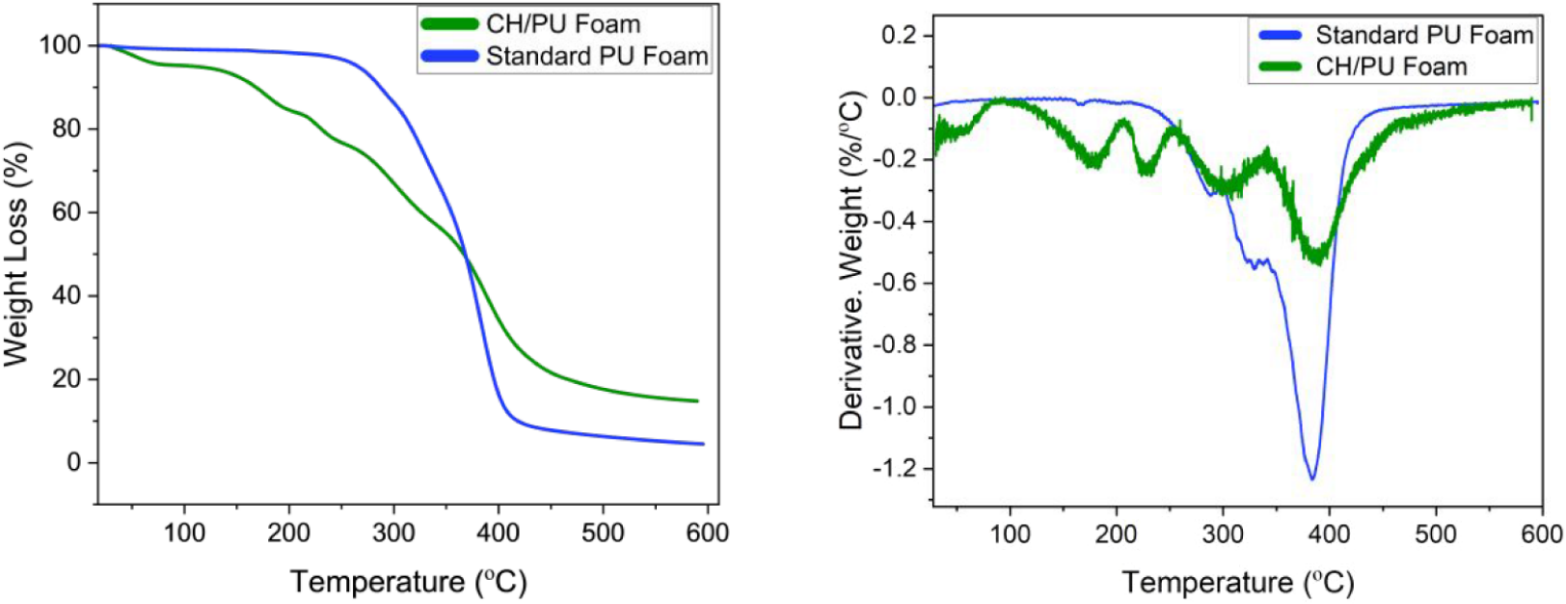
TGA/DTG curves for standard PU and CH/PU foam samples.

The thermal properties of polymeric degradation in the structure generally occur in a two-stage process. The first degradation stage begins at approximately 230 °C and continues up to 300 °C, during which the urethane bonds forming the rigid segments in the polyurethane structure are broken, resulting in the formation of liquid tar. In the second stage, structures composed of isocyanate and polyol chains undergo thermal degradation by converting into smaller molecules such as carbon oxides, amines, alcohols, ketones, aldehydes, and ethers as the temperature increases (up to 390 °C).^48^ This behavior is in comprehensive agreement with that of the standard PU sample.

According to the thermogravimetric analysis (TGA/DTG) results, the degradation temperatures at T%5, T%10, and T%20 for the standard PU foam sample were 266 °C, 286 °C, and 316 °C, respectively, whereas for the CH/PU foam samples, these values occurred at lower temperatures of approximately 110 °C, 170 °C, and 230 °C, respectively. The early onset of mass loss in the CH/PU sample was primarily attributed to free CH macromolecules not incorporated into the polymer chain rather than the foam structure itself.

This can be explained as follows: Even after esterification, the −OH functional groups, which are densely present in the CH structure, form hydrogen bonds with the water molecules present in the environment.

Therefore, the initial mass losses observed at 110 and 170 °C are primarily due to the removal of these water molecules^49^ (Note: The intense −OH peaks observed in the CH/PU sample in the FTIR spectroscopy analysis support this prediction). Additionally, the possibility that this type of mass loss may be caused by the decomposition of biuret and allophonate groups, known as thermally weak bonds in polyurethane polymers, should be considered.^50^ Therefore, the majority of the approximately 20% mass loss observed at approximately 230 °C can be attributed to the simultaneous occurrence of the first degradation stage in the PU polymer structure and the decomposition of the CH structure that does not participate in polymerization.^51^

The 50% decomposition temperature of the CH/PU sample was determined to be approximately 366 °C, a value that is largely similar to the 50% decomposition temperature of the standard PU sample (≈368 °C). This result indicates that the two systems exhibited similar thermal behaviors in the intermediate decomposition region. However, the T%75 decomposition temperature of the CH/PU sample (≈429 °C) was higher than that of the standard PU sample (≈390 °C) in the advanced decomposition stages. This difference indicates that the degradation mechanism in the CH/PU system changes in the higher temperature range and that char formation may contribute to the thermal degradation process. Nevertheless, the data obtained do not indicate that the CH/PU sample exhibits superior thermal stability across all temperature ranges; rather, the system’s degradation behavior is multistage and exhibits different thermal stability characteristics depending on the temperature.

### 3.6 SEM Investigation of Foam Morphology

Morphological analyses were performed using SEM to examine the changes in the cell structure of the PU foam sample obtained after replacing 50% of the fossil-based polyether polyol with CH/citric acid-based bio polyol. The images of the test results are shown in Figure 6.

**Figure 6:**
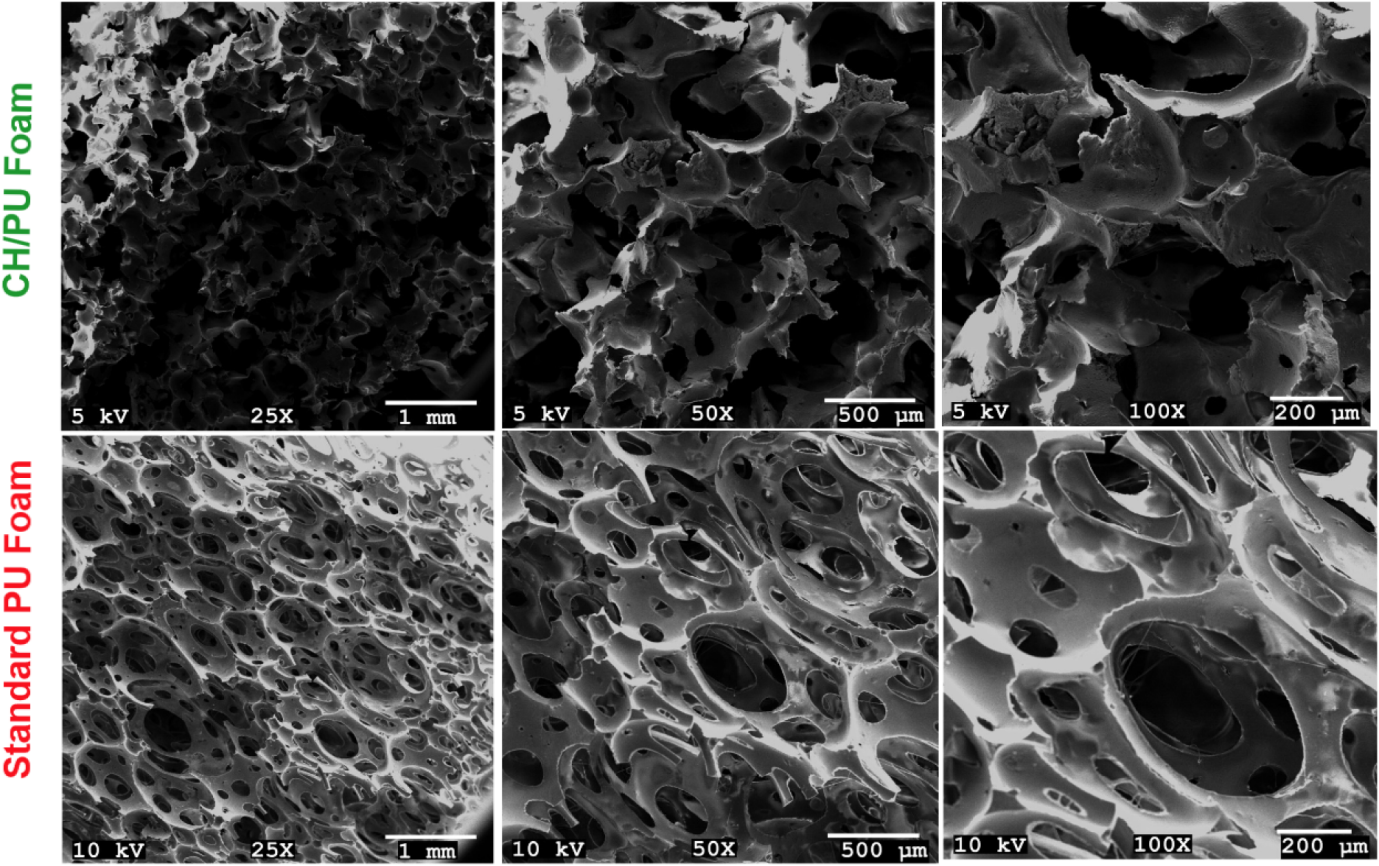
SEM images of standard PU and CH/PU foam samples.

As shown in Figure 6, the standard PU foam sample generally exhibited large nucleations with open pores and thin cell walls. In contrast, the CH/PU foam sample exhibited thickened cell walls and a structure with smaller nucleation’s and fewer open pores. This is anticipated to be due to the increased cross-link density resulting from the addition of the chitosan/citric acid-based biopolyol in the initial stage.

In addition, quantitative image analysis of the SEM micrographs presented in Figure 6 was performed using ImageJ software, and the results revealed that the number of open cells/windows observed per unit area in the Standard PU and CH/PU Foam samples was approximately 297 and 140, respectively. These findings indicate that the CH/PU Foam sample has a lower intercellular connection of open pores compared to Standard PU and exhibits a higher proportion of closed-cell morphology. This morphological change observed in CH/PU Foam suggests that the addition of chitosan-based polyol affects the system viscosity and reaction kinetics, thereby limiting the rupture of cell walls during the reaction and contributing to the formation of a pore morphology characterized by a more compact, less interconnected, and predominantly closed-cell structure.^51^

With the esterification reaction of the −OH groups of chitosan with citric acid, the total amount of OH in the environment doubles because of the contribution of the −OH group in the structure of citric acid.^52^ Thus, excess −OH groups react rapidly with NCO groups, increasing the degree of cross-linking,^53^ while the −OH groups that do not participate in the initial gelation reaction form hydrogen bonds among themselves, increasing the viscosity of the gel structure.^54^

Consequently, while standard PU foam has a more flexible structure, CH/PU foam exhibits a partially more rigid structure.

### 3.7 Apparent Density Test

The apparent density of polyurethane foams defines the mass per unit volume of the material and provides basic information regarding the cell structure and porosity of the foam. This parameter is directly related to properties such as mechanical strength, thermal conductivity, and flame-retardant performance, making it critically important for evaluating production quality and application suitability.^55,56^ In the visible density tests conducted, the density of the standard PU foam was determined to be 0.0835 g/cm^3^ (as shown in Table 6). When the polyether polyol content in the PU foam was replaced with chitosan-based polyol at a ratio of 50%, this value increased to 0.1435 g/cm^3^. This increase corresponded to approximately 71.8%. The high density of the CH/PU foam is attributed to the high number of cross-links in the structure.^53^ Additionally, the effect of hydrogen bonds should not be overlooked.^54^

**Table 6:** UL 94 Vertical Burning Test, Apparent Density, and Water Absorption Results for Standard PU and CH/PU Foam Samples

Sample	Dripping (Yes/No)	Self-Extinguishing (Yes/No)	Burning Classification (V)	Apparent Density (g/cm^3^)	Water Absorbtion (%)
Standard PU Foam	Yes	No	V2	0.0835 ± 0.0002	748.1
CH/PU Foam	Yes	No	V2	0.1435 ± 0.0003	421.5

### 3.8 UL 94 Vertical Burning Test

After using 50% chitosan/citric acid-based polyol, the UL 94 vertical burning test was applied to examine changes in the burning behavior of the foam. The obtained data are presented in Table 6. The results indicate that the combustion behavior of CH/PU foam containing 50% chitosan/citric acid-based polyol is similar to that of standard PU foam.

The liquid dripping behavior and self-extinguishing property observed in the CH/PU foam sample after flame removal were similar to those of the standard PU foam sample.

### 3.9 Water Absorption

The investigation of the water absorption behavior of PU foams enables important conclusions to be drawn regarding the material’s long-term performance, mechanical integrity, morphological structure, and cellular organization.^57^ Accordingly, samples of Standard PU and CH/PU foam were immersed in water for 72 h at 23 °C under ambient conditions to determine their water absorption properties. The experimental data are presented in Table 3. The experimental results revealed that the standard polyurethane foam containing a fossil-derived polyol exhibited a very high water absorption capacity of 748.1%. In contrast, the water absorption in the CH/PU sample, in which the polyol composition was modified with 50% biobased polyol, decreased to 421.5%. This indicates that the biobased content significantly reduces the interaction of the material with water. The improvement in the hydrophobic properties observed with an increase in the proportion of biobased polyol in the PU matrix is attributed to structural changes. In particular, the reduction in the open-cell structure^57,58^ and the denser three-dimensional network structure^59,60^ emerge as the primary factors limiting the penetration of water molecules into the foam. A more densely cross-linked structure restricts the mobility of the polymer chains, thereby hindering water diffusion.

This phenomenon can be explained in terms of the structural and chemical interactions. The polar functional groups (C=O and N–H) present in the standard PU structure tend to form hydrogen bonds with water molecules, and these interactions particularly enhance water absorption in soft segments.^61–63^ Additionally, the open-cell morphology allows water to easily penetrate the foam matrix via capillary effects and be retained within the cell voids. In contrast, soft segments exhibiting a more ordered (crystalline-like) structure or possessing a high cross-linking density cause the polymer network to become more compact and less permeable. This situation leads to the structure acquiring a relatively hydrophobic character, thereby limiting the adhesion and diffusion of water molecules within the matrix.^64^ The FTIR spectra presented in Figure 4 also support this conclusion. The band shifts associated with the distinct hydrogen bond interactions observed in the CH/PU foam sample indicate more intense intermolecular interactions within the system. Consequently, the tighter and more closed cellular/molecular network structure formed in the CH/PU foam containing the biobased polyol led to a decrease in the water absorption capacity.

### 3.10 Compression Test

Figure 7 shows the compressive stress–strain curves measured for the Standard PU and the CH/PU samples. The measured compressive strength values are 55.4 kPa for the Standard PU and 353.9 kPa for the CH/PU. The stress–strain curves have the following characteristic differences: the CH/PU curve displays a much steeper initial slope (higher initial stiffness/apparent compressive modulus), a substantially higher plateau stress, and a higher stress level before densification.

**Figure 7:**
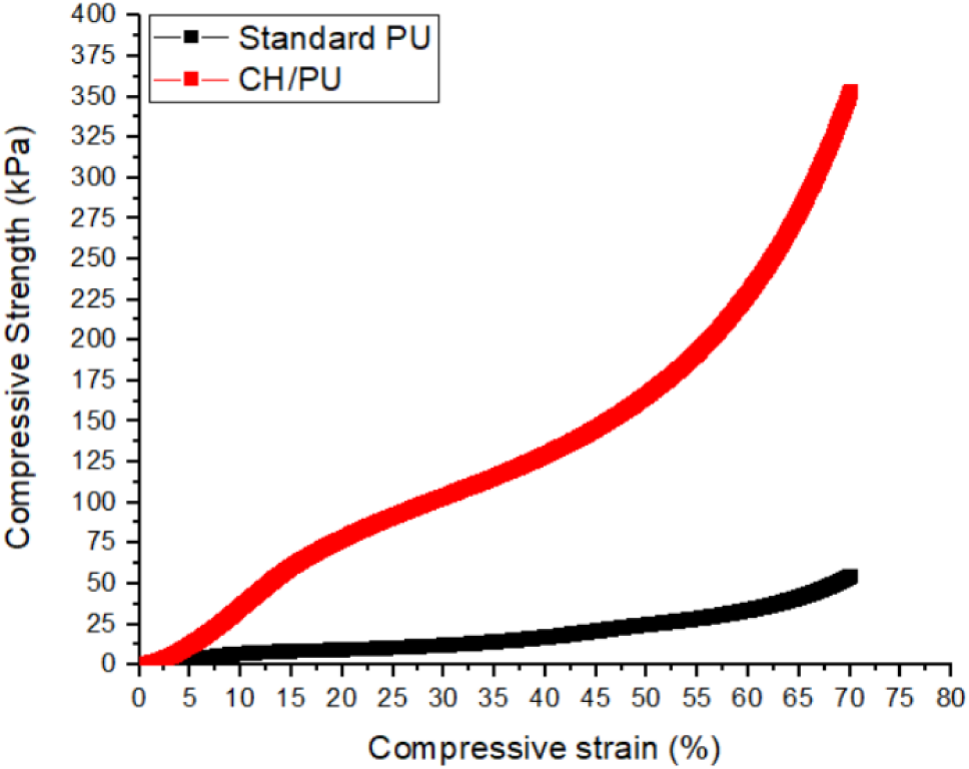
Stress–strain response under compression for standard PU and CH/PU foams.

In contrast, the standard PU shows a lower initial slope, a longer and lower plateau region typical of more compliant, thin-walled cellular foams. These qualitative curve features are consistent with the much higher load capacity of the CH/PU sample and the different cell morphologies observed by SEM. Smaller, more uniform cells with thicker walls concentrate and distribute compressive loads more effectively and reduce local buckling of cell walls; Youming et al.^65^ report that reductions in cell size and increases in wall thickness increase both modulus and compressive strength. This microstructural change therefore provides a direct micromechanical explanation for the observed strengthening. Higher cross-linking raises the rigidity of the polymer skeleton forming the cell walls and reduces local deformation, producing higher compressive stress at a given strain. Literature on chitosan-derived polyols and biobased polyurethanes reports similar trends: chitosan-containing formulations often show increased stiffness/strength (and modified thermal behavior) when incorporated appropriately into PU networks.^23^ Slower gas expansion combined with faster gelation (from high OH content) produces smaller, less open cells and higher apparent density—a processing pathway that links the chemistry (high −OH, hydrogen bonding, faster gel) to the morphological and mechanical outcomes observed. This mechanism (reduced expansion/faster gelation → higher density → higher strength) is supported by reports on biopolyol modified foams.^66^

## 4 Conclusion

MALDI-TOF MS and FTIR analyses conducted to characterize the biobased polyol demonstrated that chitosan can be liquefied through chemical modification in the presence of citric acid and glycerin, making it suitable for use in the production of biobased polyols. MALDI-TOF MS revealed that the biobased chitosan-derived polyol exhibited a narrower molecular weight distribution than pure chitosan. This indicates that the chitosan chains underwent controlled esterification reactions via citric acid and glycerin, leading to the formation of more ordered polyol structures.

FTIR analysis confirmed this chemical modification through the enhancement of ester carbonyl (C=O) bands and changes in the −OH groups. These findings indicate that the hydroxyl groups in the chitosan structure were esterified with citric acid, resulting in the formation of a modified polyol structure in conjunction with glycerin.

SEM analyses revealed that the addition of biobased polyol significantly affected the foam morphology. The images show a reduction in the open cell structure and the formation of a more closed and compact cell structure. This morphological change indicates that the cell walls of the foam structure thickened and the overall structure became more regular.

Compressive strength tests conducted to assess the mechanical properties revealed that the addition of biobased polyol significantly increased the compressive strength of the foams, consistent with the morphological changes observed in the SEM analyses. The cell structure became more closed and compact, which increased the load-bearing capacity and contributed to the improved mechanical performance.

According to the TGA results, the biobased polyol content reduced the initial decomposition temperatures. This is attributed to the hydrophilic nature of chitosan-derived structures and their segments, which have lower thermal stability. Conversely, the fact that the CH/PU foam reaches higher values at the T%75 decomposition temperature than standard PU indicates the partial retarding effect of the char layer formed at high temperatures on decomposition.

Overall, the results indicate that chitosan-based biobased polyols are a suitable alternative raw material for sustainable polyurethane production and exert significant effects on both the morphological, mechanical, and thermal properties.

## Data Availability

The data supporting the findings of this study are available within the article.
